# Differential chemokine alteration in the variants of primary progressive aphasia—a role for neuroinflammation

**DOI:** 10.1186/s12974-021-02247-3

**Published:** 2021-10-03

**Authors:** Aitana Sogorb-Esteve, Imogen J. Swift, Ione O. C. Woollacott, Jason D. Warren, Henrik Zetterberg, Jonathan D. Rohrer

**Affiliations:** 1 UK Dementia Research Institute at University College London, UCL Queen Square Institute of Neurology, University College London, London, UK; 2 Dementia Research Centre, Department of Neurodegenerative Disease, UCL Queen Square Institute of Neurology, University College London, London, WC1N 3BG UK; 3 Department of Psychiatry and Neurochemistry, The Sahlgrenska Academy at the University of Gothenburg, Mölndal, Sweden; 4 Clinical Neurochemistry Laboratory, Sahlgrenska University Hospital, Mölndal, Sweden

**Keywords:** Frontotemporal dementia, Primary progressive aphasia, Chemokines

## Abstract

**Background:**

The primary progressive aphasias (PPA) represent a group of usually sporadic neurodegenerative disorders with three main variants: the nonfluent or agrammatic variant (nfvPPA), the semantic variant (svPPA), and the logopenic variant (lvPPA). They are usually associated with a specific underlying pathology: nfvPPA with a primary tauopathy, svPPA with a TDP-43 proteinopathy, and lvPPA with underlying Alzheimer’s disease (AD). Little is known about their cause or pathophysiology, but prior studies in both AD and svPPA have suggested a role for neuroinflammation. In this study, we set out to investigate the role of chemokines across the PPA spectrum, with a primary focus on central changes in cerebrospinal fluid (CSF)

**Methods:**

Thirty-six participants with sporadic PPA (11 svPPA, 13 nfvPPA, and 12 lvPPA) as well as 19 healthy controls were recruited to the study and donated CSF and plasma samples. All patients with lvPPA had a tau/Aβ42 biomarker profile consistent with AD, whilst this was normal in the other PPA groups and controls. We assessed twenty chemokines in CSF and plasma using Proximity Extension Assay technology: CCL2 (MCP-1), CCL3 (MIP-1a), CCL4 (MIP-1β), CCL7 (MCP-3), CCL8 (MCP-2), CCL11 (eotaxin), CCL13 (MCP-4), CCL19, CCL20, CCL23, CCL25, CCL28, CX3CL1 (fractalkine), CXCL1, CXCL5, CXCL6, CXCL8 (IL-8), CXCL9, CXCL10, and CXCL11.

**Results:**

In CSF, CCL19 and CXCL6 were decreased in both svPPA and nfvPPA compared with controls whilst CXCL5 was decreased in the nfvPPA group with a borderline significant decrease in the svPPA group. In contrast, CCL2, CCL3 and CX3CL1 were increased in lvPPA compared with controls and nfvPPA (and greater than svPPA for CX3CL1). CXCL1 was also increased in lvPPA compared with nfvPPA but not the other groups. CX3CL1 was significantly correlated with CSF total tau concentrations in the controls and each of the PPA groups. Fewer significant differences were seen between groups in plasma, although in general, results were in the opposite direction to CSF, i.e. decreased in lvPPA compared with controls (CCL3 and CCL19), and increased in svPPA (CCL8) and nfvPPA (CCL13).

**Conclusion:**

Differential alteration of chemokines across the PPA variants is seen in both CSF and plasma. Importantly, these results suggest a role for neuroinflammation in these poorly understood sporadic disorders, and therefore also a potential future therapeutic target.

**Supplementary Information:**

The online version contains supplementary material available at 10.1186/s12974-021-02247-3.

## Background

The primary progressive aphasias (PPA) are a rare group of disorders characterised by focal degeneration of the brain regions involved in language function [1] and fall within the frontotemporal dementia (FTD) spectrum. There are three main variants—the nonfluent or agrammatic variant (nfvPPA), the semantic variant (svPPA), and the logopenic variant (lvPPA)—distinguished by the language deficits with which they present as well as their neuroanatomical signatures [2]. Similarly, the predominant neuropathology underlying each of the variants usually differs, with nfvPPA most commonly a tauopathy such as progressive supranuclear palsy or corticobasal degeneration [3, 4], whilst svPPA is almost always a TDP-43 proteinopathy [5]. In contrast, lvPPA is not a frontotemporal lobar degeneration (FTLD) pathologically (i.e. not a primary tauopathy or TDP-43 proteinopathy) in most cases [6]; instead, the underlying pathology is usually that of Alzheimer’s disease (AD), and lvPPA is often therefore considered an atypical language variant of AD.

Recent studies have shown that neuroinflammation is an important pathophysiological factor in neurodegenerative disorders [7]. Although less research has been performed in FTD, molecular, pathological, and biomarker studies all suggest that inflammation is important here as well (reviewed in Bright et al. [8]). The process of neuroinflammation is complex and multistage but involves activation of glial cells, which in turn leads to upregulation of several proteins that help to guide the response. These include chemokines, a family of proteins that regulate leukocyte traffic but also have a number of other roles both within the immune system and outside, e.g. in development and synaptic transmission [9]. Only a few studies have so far investigated changes in chemokines in FTD spectrum disorders  [10, 11], and so we aimed to assess this more thoroughly by using a panel of chemokines in the biofluids of a well-defined cohort of people from across the PPA spectrum, particularly focusing on changes centrally within the cerebrospinal fluid (CSF).

## Methods

### Participants

Thirty-six people with sporadic PPA and available CSF and plasma were consecutively recruited through the Longitudinal Investigation of FTD (LIFTD) study at University College London (Table 1): 11 svPPA, 13 nfvPPA, and 12 lvPPA, diagnosed according to current consensus criteria [2]. All cases were negative for a C9orf72 expansion and mutations in any of the genes causative of FTD. 19 healthy controls were recruited over the same time period. All patients with lvPPA had a biomarker profile consistent with underlying Alzheimer’s disease: mean (standard deviation) total tau/Aβ42 ratio (INNOTEST®, Fujirebio Europe N.V., Gent, Belgium) of 3.2 (2.2) with a range 1.2 to 8.3 where > 1 is considered abnormal. All svPPA or nfvPPA participants and all controls had a ratio < 1.

**Table 1:** Demographics of participants in the study. PPA, primary progressive aphasia; svPPA, semantic variant; nfv, nonfluent variant; lv, logopenic variant. N, number of participants. Values are shown as mean (standard deviation)

	Controls	svPPA	nfvPPA	lvPPA
*N*	19	11	13	12
Age at CSF	63.5 (6.9)	60.5 (5.9)	67.0 (6.3)	66.7 (6.3)
Sex (% male)	47.4	54.5	53.8	50.0
Disease duration at CSF	N/A	4.6 (2.0)	4.5 (1.9)	3.6 (2.2)
Aβ42	999.9 (235.4)	879.7 (259.5)	845.6 (318.3)	439.8 (159.4)
Total tau	325.7 (93.3)	355.7 (152.9)	405.8 (184.7)	1206 (555.4)
Total tau/Aβ42 ratio	0.3 (0.1)	0.4 (0.1)	0.5 (0.3)	3.2 (2.2)

### Proximity Extension Assay panel

Twenty chemokines were measured in the CSF and plasma of all participants using Proximity Extension Assay technology on the Olink® neuroinflammatory panel [12]. Briefly, samples were incubated with matched antibodies with DNA tags. When matched antibodies come in close proximity, DNA tags will only hybridise when the coupled antibodies match and then the sequence is amplified by qPCR. Results are expressed as normalised protein expression values. The chemokines measured were CCL2 (MCP-1), CCL3 (MIP-1a), CCL4 (MIP-1β), CCL7 (MCP-3), CCL8 (MCP-2), CCL11 (eotaxin), CCL13 (MCP-4), CCL19, CCL20, CCL23, CCL25, CCL28, CX3CL1 (fractalkine), CXCL1, CXCL5, CXCL6, CXCL8 (IL-8), CXCL9, CXCL10, and CXCL11.

### Statistical analysis

All statistical analyses were performed in STATA (v.16)) with the primary analysis focused on the results in the CSF. As this was an exploratory study, no correction for multiplicity was performed. The Shapiro-Wilk test was performed to determine the normality of distribution of each chemokine measure in each group.

Within the control group, Spearman correlation coefficients were assessed for each individual chemokine between their values and the age of participants at CSF collection. Sex differences were calculated using Mann-Whitney *U* tests.

The levels of each chemokine in the CSF were then compared between groups using a linear regression model; bootstrapping with 1000 repetitions was used if the chemokine measures were not normal.

Spearman correlation coefficients were assessed for each individual chemokine between their values and each of Aβ42 and total tau (t-tau) concentrations within the control and PPA groups.

In order to explore the relationship between chemokine values in CSF and plasma, Spearman correlation coefficients were assessed in each individual chemokine between CSF and plasma values within the control group as well as the disease groups.

The levels of each chemokine in the plasma were subsequently compared between groups using a linear regression model as previously; bootstrapping with 1000 repetitions was used if the chemokine measures were not normal.

## Results

There were no significant differences between the groups in terms of either age at CSF collection or sex (Table 1).

Within the control group, no significant correlations were found between chemokines and age except for CXCL9 (rho = 0.55, *p* = 0.016) (Supplementary Table 1). No significant differences were found between chemokine values in males and females except in CCL28 where concentrations were lower in females (0.4 (0.1) versus 0.5 (0.1) in males, *p* = 0.034).

**Fig. 1:**
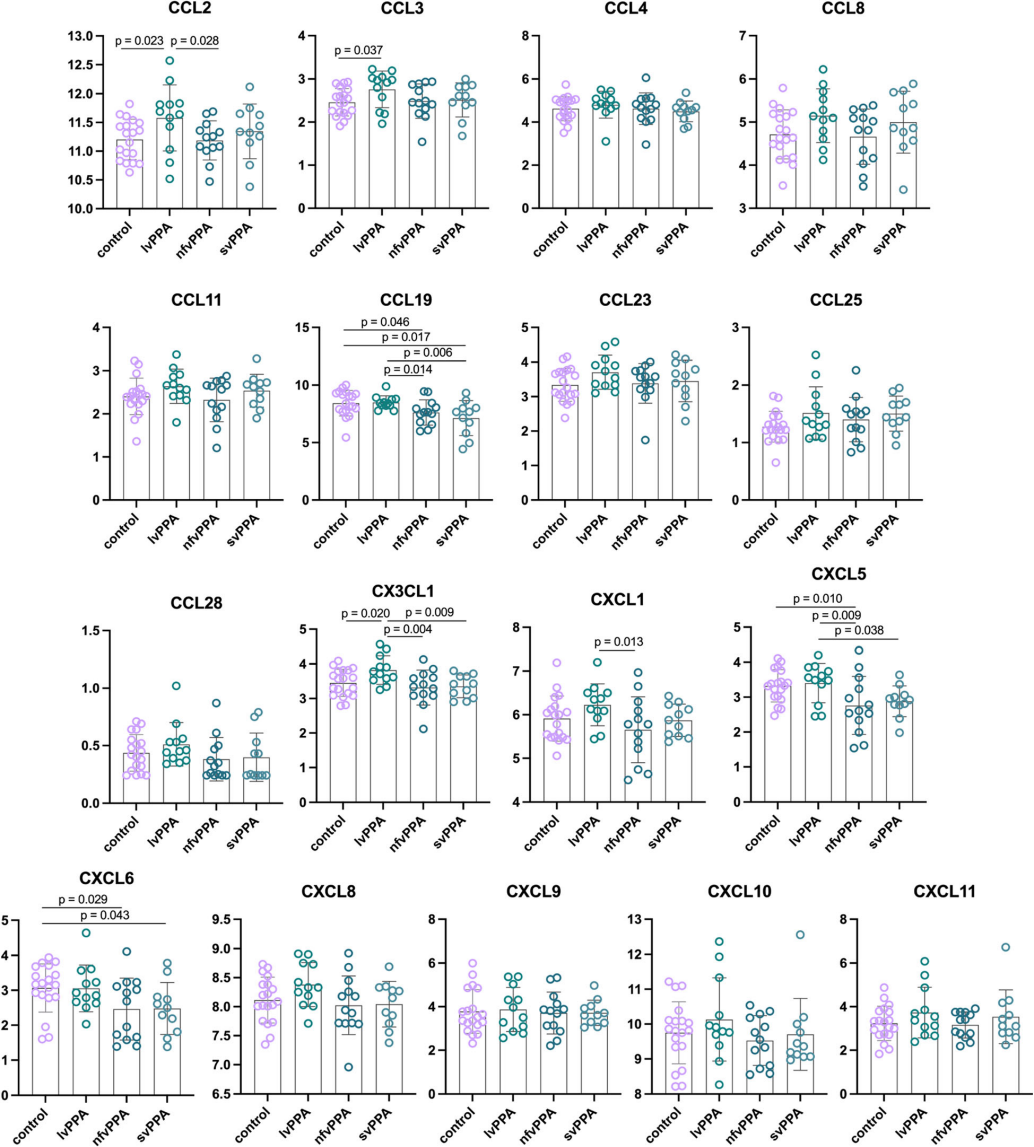
Mean normalised protein expression values for the chemokines in controls and each of PPA groups in CSF. Significant differences with *p* values are shown on the graphs

Within CSF, three chemokines had > 80% of values across the groups below the lower limit of detection (CCL20 44/55, CCL13 53/55, CCL7 54/55) and were not assessed further. Seven of the remaining 17 chemokines showed a significant difference between groups. In both svPPA and nfvPPA, CCL19 and CXCL6 were decreased compared with controls (Fig. 1, Supplementary Table 2). CCL19 was additionally significantly decreased in both nfvPPA and svPPA compared with lvPPA, as was CXCL5, although this chemokine was decreased only in the nfvPPA group compared with controls (albeit with a borderline significant decrease in the svPPA group). In contrast, CCL2, CCL3 and CX3CL1 were increased in lvPPA compared with controls (and greater than nfvPPA for CCL2, and both nfvPPA and svPPA for CX3CL1). CXCL1 was also increased in lvPPA compared with nfvPPA but not the other groups.

No correlations were found between Aβ42 concentrations and values of any of the chemokines within the controls or the PPA groups except for a correlation in the nfvPPA group for CXCL6: rho = 0.58, *p* = 0.033 (Supplementary Figure 1). However, CX3CL1 was significantly correlated with t-tau concentrations in the controls and each of the PPA groups (Supplementary Figure 1): controls rho = 0.51, *p* = 0.025, svPPA rho = 0.64, *p* = 0.035, nfvPPA rho = 0.72, *p* = 0.005, lvPPA rho = 0.59, *p* = 0.045. None of the other chemokines correlated with t-tau across the groups but CCL4 was correlated in controls (rho = 0.59, *p* = 0.033), and CXCL5 was correlated in svPPA (rho = 0.65, *p* = 0.029).

In the chemokines that were found to be abnormal in CSF across groups, none of the CSF values were correlated with plasma values within the control group or any of the PPA groups. Only chemokines for which no significant group differences were seen showed a correlation between CSF and plasma values, both within the control group (CCL8: rho = 0.48, *p* = 0.039; CCL11: rho = 0.46; *p* = 0.048; CCL25: rho = 0.53, *p* = 0.021), the lvPPA group (CCL4: rho = 0.68, *p* = 0.015; CXCL8: rho = 0.79; *p* = 0.002), and the nfvPPA group (CCL4: rho = 0.78, *p* = 0.002; CCL11: rho = 0.64; *p* = 0.017; CCL25: rho = 0.58, *p* = 0.040; CXCL10: rho = 0.66, *p* = 0.014). No correlations were seen in the svPPA group.

Within plasma, fewer significant differences were seen between groups (Supplementary Figure 1, Supplementary Table 3). However, CCL8 was higher in svPPA compared with controls and CCL13 was higher (with CCL20 lower) in nfvPPA compared with controls. In contrast, CCL3 and CCL19 were significantly lower in lvPPA compared with controls and the svPPA group (as well as the nfvPPA group for CCL3). Additionally, CCL4 was lower in lvPPA compared with nfvPPA but not with the other groups.

## Discussion

We report chemokine levels in biofluids in PPA, showing differential alteration across the PPA variants. Interestingly, the direction of significant change differed between those with underlying AD pathologically (lvPPA, where CSF values were increased and plasma values decreased) and those with FTLD (svPPA and nfvPPA, where CSF values were decreased, and for all but one chemokine, plasma values were increased). Comparing the CSF chemokine values with CSF biomarkers of amyloid and tau, CX3CL1, which was increased in lvPPA, was significantly correlated with t-tau concentrations across all of the groups.

In keeping with lvPPA being associated with underlying AD pathology, the findings of increased CCL2, CCL3, and CX3CL1 in this group parallel previous studies in those with a typical AD clinical presentation [13–18]. CCL2 (MCP-1) is expressed by neurons, astrocytes, and microglia and has previously been shown to be raised in the CSF of people with typical AD including those with mild cognitive impairment (MCI) [13, 14, 19]. A further study also showed that CSF CCL2 concentrations correlated with the extent of brain atrophy and cognitive impairment in AD [15], with one report suggesting that higher levels were associated with more rapid progression from MCI to AD [16]. CCL3 (MIP-1a) is also expressed by neurons, astrocytes, and microglia [20], and this expression has been shown to be increased in the brains of people with AD as well as in mouse models [21, 22]. Here, we show increased levels of CCL3 in the CSF of the atypical AD phenotype lvPPA. Interestingly, we also show lower levels of CCL3 peripherally in the plasma, a finding also previously found by another group in serum [17]. The pathophysiological explanation for this difference between central and peripheral CCL3 levels remains unclear. Lastly, CX3CL1 (fractalkine) is produced by neurons, particularly in the hippocampus and cortex, and suppresses microglial activation [23]. Importantly, from an AD perspective, CX3CL1 has also been shown to be upregulated in the hippocampus during memory-associated synaptic plasticity [23]. Several studies have demonstrated significantly higher CSF concentrations of CX3CL1 in people with MCI and AD [21], with concentration being shown to differentiate MCI from controls with high sensitivity and specificity [18]. Our study also showed a strong correlation with t-tau in lvPPA as well as in the controls and other groups, suggesting an association with increased neuronal damage particularly in the AD brain.

Although non-significant, a number of other chemokines showed a trend to an increase in lvPPA compared with controls, e.g. CCL23 (*p* = 0.068), increased concentrations of which have previously been shown to help predict progression from MCI to AD [24]. CXCL1 showed a significant increase in lvPPA compared with nfvPPA; this chemokine has also been shown to be increased in the CSF of people with AD in a prior study [25].

Overall, this study highlights the important pathophysiological overlap of atypical variants of AD with the more typical variant. Here, we show that the central chemokine profile of change is similar in lvPPA to that found in amnestic AD.

In contrast, the two FTLD pathological forms of PPA show differential alterations of chemokines to lvPPA, with parallel decreases in CCL19, CXCL5, and CXCL6 in both svPPA and nfvPPA. This finding suggests that these are related to underlying FTLD pathology per se, rather than the individual proteinopathy, as svPPA (TDP-43) and nfvPPA (tau) usually differ in their primary pathological cause. Unfortunately, little is known about alterations in chemokines levels in FTD so far. Only a few small studies have investigated chemokines, showing decreased levels of CCL5 (RANTES) and increased levels of CCL2, CXCL8, and CXCL10 in CSF [8, 10]. Previous studies have not investigated such an extensive set of chemokines in FTD across differential pathologies. CCL19, CXCL5, and CXCL6 are all expressed in the brain, although their roles are not clear. Nonetheless, CCL19 in particular has been studied in neuroinflammation-related diseases such as multiple sclerosis [26, 27], although here the levels were increased rather than decreased as found in our study. Whilst these are preliminary findings in nfvPPA and svPPA, they are important in signalling a role for neuroinflammation in these disorders. Little is currently known about why these sporadic diseases develop, and although a few studies have previously suggested a role for neuroinflammation in svPPA [28, 29], this study also suggests it is a relevant pathophysiological phenomenon in nfvPPA also. Interestingly, the levels of chemokines are generally decreased in svPPA and lvPPA compared to the increase seen in lvPPA—the reason for this is not clear: the inflammatory response to neurodegeneration is complex and studies investigating the response to inflammation including the involvement of the resolution pathway will be important.

Whilst central neuroinflammatory processes in neurodegenerative disorders are increasingly well-studied, fewer studies have investigated peripheral immune findings. There was generally poor correlation between CSF chemokine values and those in plasma within the controls, suggesting that peripheral values generally do not represent central levels. However, abnormal values were found in each of the PPA variants compared with controls (and for all but one measure, in the opposite direction to that found centrally in CSF). Certainly for svPPA, a previous study has showed an increase in systemic autoimmune disease compared with controls [29], but otherwise, little is known about peripheral inflammation in PPA, and more studies are required.

There are a number of limitations of the study. We did not have access to detailed behavioural or neuropsychometry data within the cohort, and it would be useful for future studies to investigate the correlation of clinical features with chemokine levels. The presence of co-morbidities such as systemic disease, mood disorders, and cerebrovascular disease (including for the latter, the presence of white matter hyperintensities on MRI) were also not evaluated: their effect on chemokine levels would be important to clarify in further analyses. Whilst each group was of similar disease duration, participants were on average around 3 to 5 years into their illness. It would therefore be helpful to study both people very early in their clinical syndrome as well as to investigate longitudinal change in chemokines in PPA in order to understand the temporal relationship within the disease.

## Conclusions

Overall, this study highlights the complex inflammatory response in the different variants of PPA and shows clear differences between those with AD and FTLD pathology. Such biomarkers may be helpful in future trials for a number of reasons including assessing the extent of neuroinflammation present [30], although not for individually classifying the different forms of PPA. Our results establish a baseline for further study of the role of chemokines in PPA with the potential role of neuroinflammation as a therapeutic target in these sporadic disorders an important area of future research.

## Supplementary Information

**Additional file 1: Supplementary Figure 1.** Mean normalized protein expression values for the chemokines in controls and each of PPA groups in plasma. Significant differences with *p* values are shown on the graphs.

**Additional file 2: Supplementary Table 1.** Spearman correlation coefficients and *p*-values comparing chemokines with age at CSF collection within the control group. **Supplementary Table 2.** Mean (standard deviation) normalized protein expression values for the chemokines in controls and each of PPA groups in CSF. Mean differences between the PPA groups and controls along with 95% confidence intervals and *p* values (significant in bold) are shown underneath. N/A = not assessed >80% values were below the lower limit of detection. **Supplementary Table 3.** Mean (standard deviation) normalized protein expression values for the chemokines in controls and each of PPA groups in plasma. Mean differences between the PPA groups and controls along with 95% confidence intervals and *p* values (significant in bold) are shown underneath. N/A = not assessed >80% values were below the lower limit of detection.

## Data Availability

The datasets used and/or analysed during the current study are available from the corresponding author on reasonable request.

## Abbreviations

PPA: Primary progressive aphasia
FTD: Frontotemporal dementia
nfvPPA: Nonfluent/agrammatic variant PPA
svPPA: Semantic variant PPA
lvPPA: Logopenic variant PPA
FTLD: Frontotemporal lobar degeneration
TDP-43: TAR DNA-binding protein 43
AD: Alzheimer’s disease
CSF: Cerebrospinal fluid
C9orf72: Chromosome 9 open reading frame 72
qPCR: Quantitative Polymerase Chain Reaction
CCL2 (MCP-1): C-C motif chemokine 2 (Monocyte chemoattractant protein 1)
CCL3 (MIP-1a): C-C motif chemokine 3 (Macrophage inflammatory protein 1 alpha)
CCL4 (MIP-1β): C-C motif chemokine 4 (Macrophage inflammatory protein 1 beta)
CCL7 (MCP-3): C-C motif chemokine 7 (Monocyte chemoattractant protein 3)
CCL8 (MCP-2): C-C motif chemokine 8 (Monocyte chemoattractant protein 2)
CCL11: Eotaxin
CCL13 (MCP-4): C-C motif chemokine 13 (Monocyte chemoattractant protein 4)
CCL19: C-C motif chemokine 19
CCL20: C-C motif chemokine 20
CCL23: C-C motif chemokine 23
CCL25: C-C motif chemokine 25
CCL28: C-C motif chemokine 28
CX3CL1: Fractalkine
CXCL1: Growth-regulated alpha protein
CXCL5: C-X-C motif chemokine 5
CXCL6: C-X-C motif chemokine 6
CXCL8 (IL-8): Interleukin 8
CXCL9: C-X-C motif chemokine 9
CXCL10: C-X-C motif chemokine 10
CXCL11: C-X-C motif chemokine 11
MCI: Mild cognitive impairment
