# Supplementary figures and images for: Differential chemokine alteration in the variants of primary progressive aphasia—a role for neuroinflammation
Source: J Neuroinflammation. 2021 Oct 3;18:224. doi: 10.1186/s12974-021-02247-3 (PMC8489077; doi:10.1186/s12974-021-02247-3)

**CCL3**

$p = 0.014$   $p = 0.004$   
 $p = 0.041$

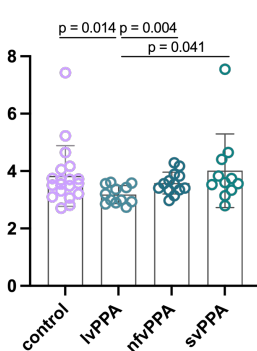**CCL4**

$p = 0.037$

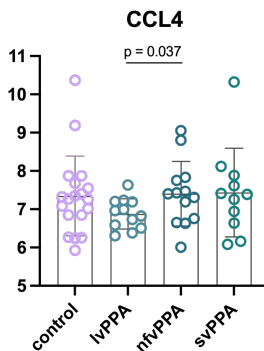**CCL7**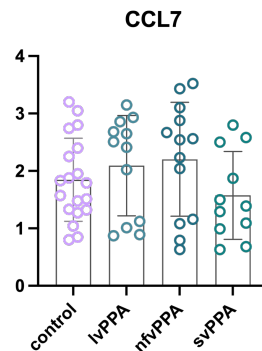**CCL8**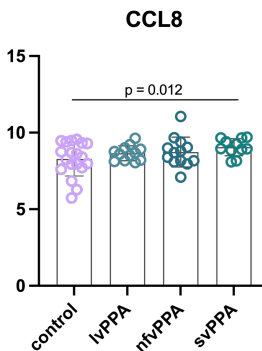**CCL2**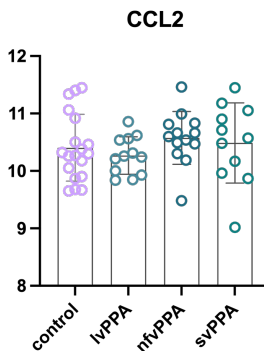**CCL11**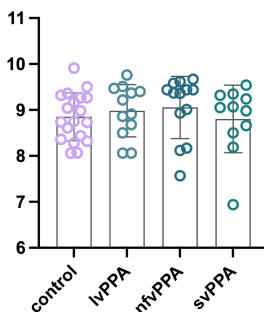**CCL13**

$p = 0.047$

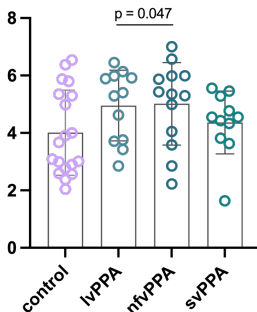**CCL19**

$p = 0.001$   $p = 0.009$

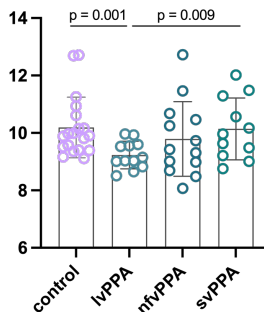**CCL20**

$p = 0.031$

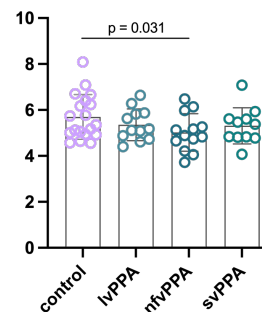**CCL23**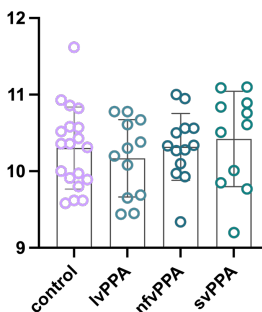**CCL25**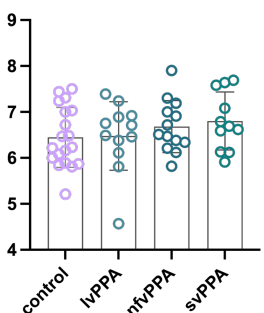**CCL28**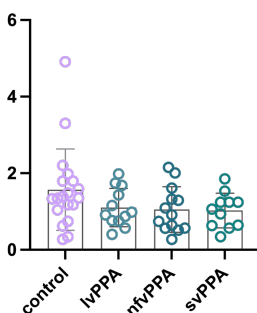**CX3CL1**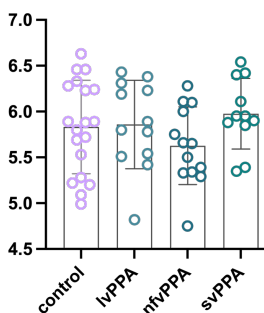**CXCL1**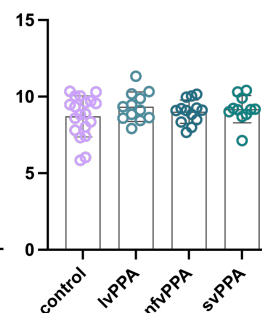**CXCL5**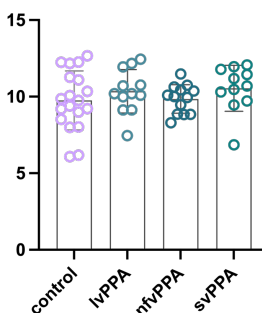**CXCL6**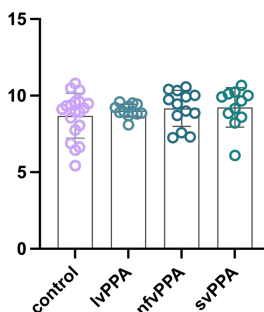**CXCL8**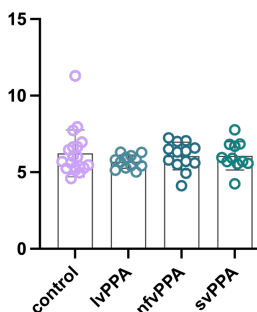**CXCL9**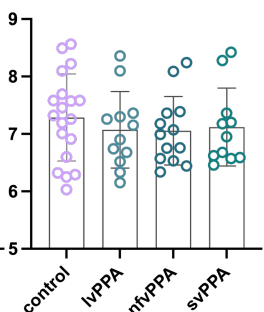**CXCL10**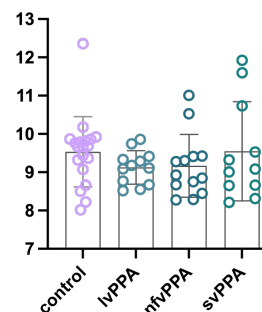**CXCL11**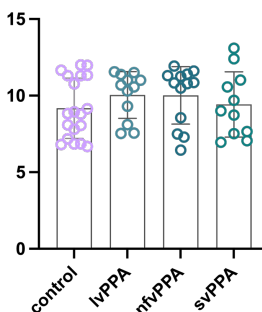

Supplement: Supplementary file 1 — Additional file 1: Supplementary Figure 1. Mean normalized protein expression values for the chemokines in controls and each of PPA groups in plasma. Significant differences with p values are shown on the graphs. [file 12974_2021_2247_MOESM1_ESM.zip › Supplemental Figure 1 (plasma).pdf]

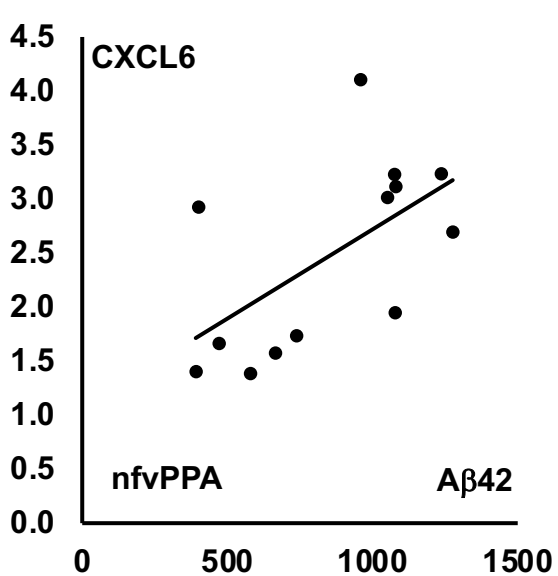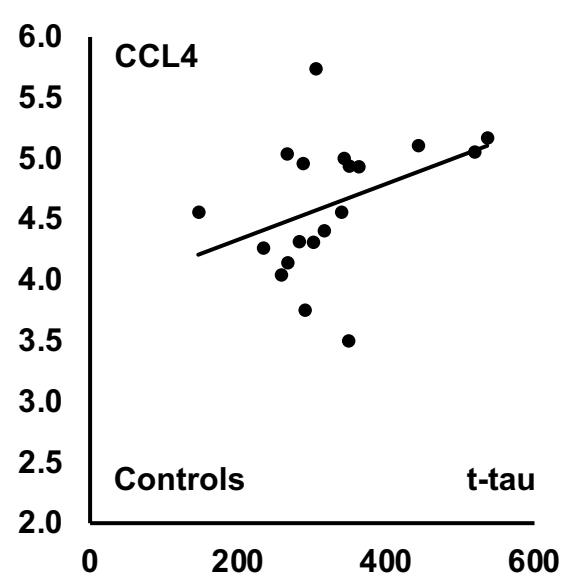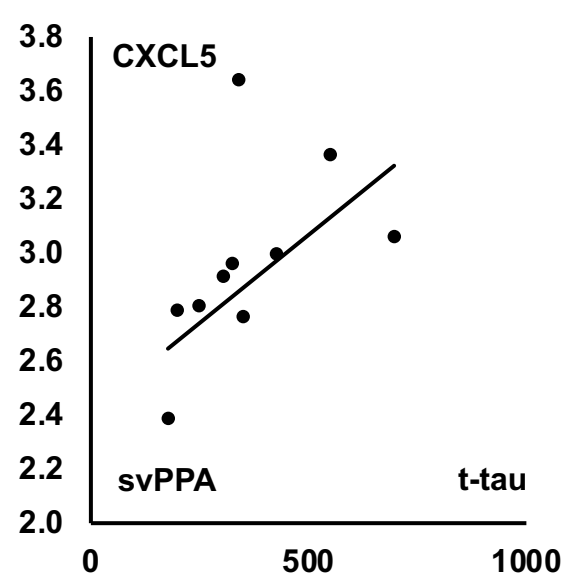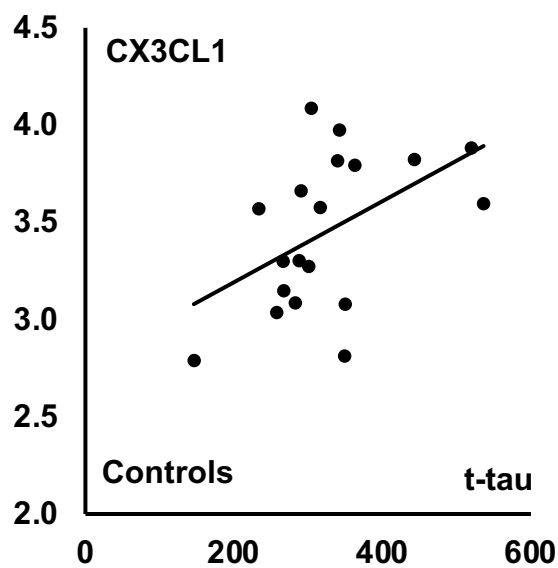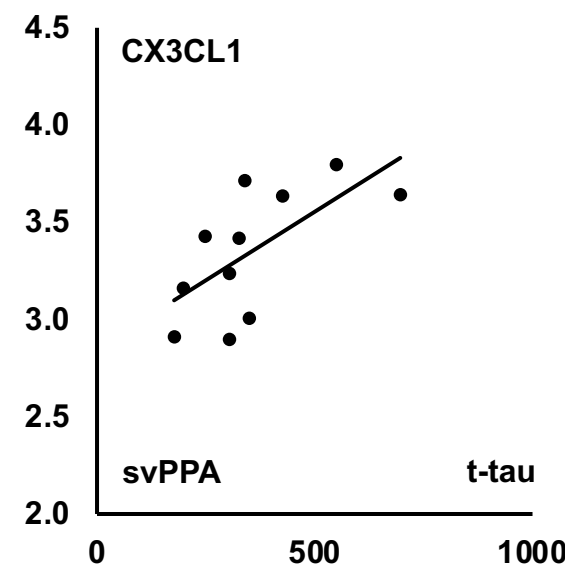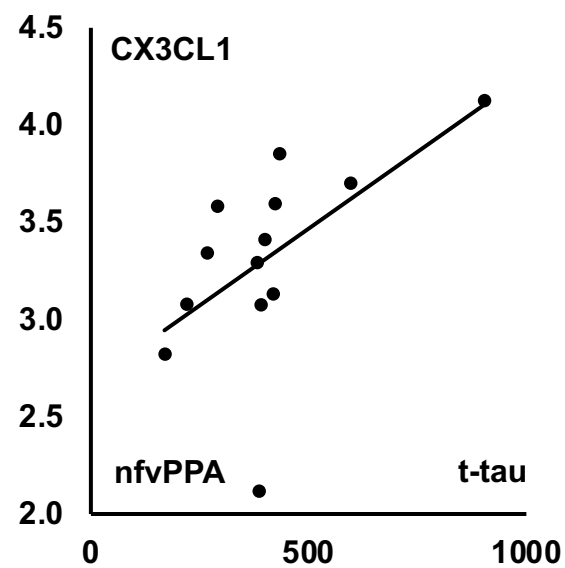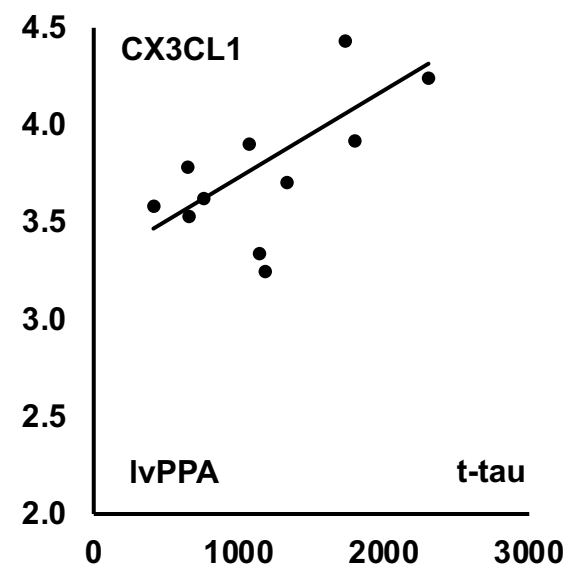

Supplement: Supplementary file 1 — Additional file 1: Supplementary Figure 1. Mean normalized protein expression values for the chemokines in controls and each of PPA groups in plasma. Significant differences with p values are shown on the graphs. [file 12974_2021_2247_MOESM1_ESM.zip › Supplementary_Figure_1.pdf]
