# Supplementary material for: Differential chemokine alteration in the variants of primary progressive aphasia—a role for neuroinflammation
Source: J Neuroinflammation. 2021 Oct 3;18:224. doi: 10.1186/s12974-021-02247-3 (PMC8489077; doi:10.1186/s12974-021-02247-3)
Supplement: Supplementary file 2 — Additional file 2: Supplementary Table 1. Spearman correlation coefficients and p-values comparing chemokines with age at CSF collection within the control group. Supplementary Table 2. Mean (standard deviation) normalized protein expression values for the chemokines in controls and each of PPA groups in CSF. Mean differences between the PPA groups and controls along with 95% confidence intervals and p values (significant in bold) are shown underneath. N/A = not assessed >80% values were below the lower limit of detection. Supplementary Table 3. Mean (standard deviation) normalized protein expression values for the chemokines in controls and each of PPA groups in plasma. Mean differences between the PPA groups and controls along with 95% confidence intervals and p values (significant in bold) are shown underneath. N/A = not assessed >80% values were below the lower limit of detection. [file 12974_2021_2247_MOESM2_ESM.docx]

**Supplementary Table 1. Spearman correlation coefficients and p-values comparing chemokines with age at CSF collection within the control group.**

|  | **rho** | **p** |
| --- | --- | --- |
| CCL2 | -0.04 | 0.866 |
| CCL3 | 0.24 | 0.320 |
| CCL4 | 0.00 | 1.000 |
| CCL8 | 0.16 | 0.498 |
| CCl11 | -0.01 | 0.957 |
| CCL19 | -0.42 | 0.075 |
| CCL23 | 0.03 | 0.900 |
| CCL25 | 0.18 | 0.449 |
| CCL28 | 0.22 | 0.366 |
| CX3CL1 | -0.38 | 0.112 |
| CXCL1 | -0.05 | 0.826 |
| CXCL5 | -0.36 | 0.127 |
| CXCL6 | -0.01 | 0.974 |
| CXCL8 | -0.04 | 0.877 |
| CXCL9 | **0.55** | **0.016** |
| CXCL10 | -0.04 | 0.866 |
| CXCL11 | 0.03 | 0.894 |

**Supplementary Table 2. Mean (standard deviation) normalized protein expression values for the chemokines in controls and each of PPA groups in CSF. Mean differences between the PPA groups and controls along with 95% confidence intervals and p values (significant in bold) are shown underneath. N/A = not assessed >80% values were below the lower limit of detection.**

|  | **Controls** | **svPPA** | **nfvPPA** | **lvPPA** |
| --- | --- | --- | --- | --- |
| CCL2 | 11.2 (0.4) | 11.3 (0.5) | 11.2 (0.3) | **11.6 (0.6)** |
|  |  | 0.14 (-0.19, 0.47)  p=0.394 | -0.02 (-0.33, 0.29)  p=0.909 | **0.37 (0.05, 0.69)**  **p=0.023** |
| CCL3 | 2.5 (0.3) | 2.5 (0.4) | 2.5 (0.4) | **2.8 (0.4)** |
|  |  | 0.06 (-0.23, 0.34)  p=0.695 | 0.03 (-0.25, 0.30)  p=0.844 | **0.30 (0.18, 0.58)**  **p=0.037** |
| CCL4 | 4.6 (0.6) | 4.5 (0.5) | 4.6 (0.7) | 4.8 (0.6) |
|  |  | -0.12 (-0.58, 0.34)  p=0.596 | 0.00 (-0.43, 0.44)  p=0.990 | 0.18 (-0.26, 0.53)  p=0.410 |
| CCL7 | N/A | N/A | N/A | N/A |
|  |  | N/A | N/A | N/A |
| CCL8 | 4.7 (0.6) | 5.0 (0.7) | 4.7 (0.6) | 5.1 (0.6) |
|  |  | 0.28 (-0.20, 0.76)  p=0.242 | -0.05 (-0.51, 0.40)  p=0.816 | 0.43 (-0.32, 0.90)  p=0.067 |
| CCL11 | 2.4 (0.4) | 2.5 (0.4) | 2.3 (0.5) | 2.6 (0.4) |
|  |  | 0.13 (-0.20, 0.46)  p-0.419 | -0.08 (-0.39, 0.23)  p=0.607 | 0.24 (-0.08, 0.56)  p=0.144 |
| CCL13 | N/A | N/A | N/A | N/A |
|  |  | N/A | N/A | N/A |
| CCL19 | 8.4 (1.1) | **7.1 (1.5)** | **7.6 (1.1)** | 8.5 (0.6) |
|  |  | **-1.30 (-2.37, -0.24)**  **p=0.017** | **-0.82 (-1.63, -0.01)**  **p=0.046** | 0.06 (-0.56, 0.69)  p=0.847 |
| CCL20 | N/A | N/A | N/A | N/A |
|  |  | N/A | N/A | N/A |
| CCL23 | 3.3 (0.5) | 3.5 (0.6) | 3.4 (0.6) | 3.7 (0.5) |
|  |  | 0.11 (-0.29, 0.51)  p=0.586 | 0.04 (-0.34, 0.43)  p=0.819 | 0.36 (-0.03, 0.76)  p=0.068 |
| CCL25 | 1.3 (0.3) | 1.5 (0.3) | 1.4 (0.4) | 1.5 (0.5) |
|  |  | 0.23 (-0.04, 0.50)  p=0.093 | 0.12 (-0.13, 0.38)  p=0.331 | 0.24 (-0.02, 0.50)  p=0.071 |
| CCL28 | 0.4 (0.2) | 0.4 (0.2) | 0.4 (0.2) | 0.5 (0.2) |
|  |  | -0.04 (-0.18, 0.11)  p=0.606 | -0.05 (-0.18, 0.07)  p=0.389 | 0.07 (-0.06, 0.21)  p=0.268 |
| CX3CL1 | 3.5 (0.4) | 3.4 (0.3) | 3.3 (0.5) | **3.8 (0.4)** |
|  |  | -0.10 (-0.42, 0.22)  p=0.523 | -0.14 (-0.44, 0.17)  p=0.370 | **0.37 (0.06, 0.68)**  **p=0.020** |
| CXCL1 | 5.9 (0.5) | 5.9 (0.4) | 5.7 (0.8) | 6.2 (0.5) |
|  |  | -0.04 (-0.46, 0.38)  p=0.852 | -0.26 (-0.66, 0.14)  p=0.206 | 0.31 (-0.95, 0.72)  p=0.129 |
| CXCL5 | 3.3 (0.5) | 2.9 (0.4) | **2.8 (0.8)** | 3.4 (0.6) |
|  |  | -0.44 (-1.99, 0.00)  p=0.052 | **-0.56 (-0.98, -0.14)**  **p=0.010** | 0.08 (-0.35, 0.51)  p=0.712 |
| CXCL6 | 3.1 (0.7) | **2.5 (0.7)** | **2.5 (0.9)** | 3.1 (0.7) |
|  |  | **-0.59 (-1.16, -0.02)**  **p=0.043** | **-0.60 (-1.14, -0.63)**  **p=0.029** | -0.13 (-0.57, 0.54)  p=0.963 |
| CXCL8 | 8.1 (0.4) | 8.0 (0.4) | 8.0 (0.5) | 8.4 (0.4) |
|  |  | -0.07 (-0.39, 0.21)  p=0.655 | -0.09 (-0.39, 0.21)  p=0.553 | 0.27 (-0.05, 0.58)  p=0.092 |
| CXCL9 | 3.8 (1.0) | 3.7 (0.6) | 3.7 (1.0) | 3.9 (1.0) |
|  |  | -0.07 (-0.77, 0.64)  p=0.848 | -0.08 (-0.75, 0.60)  p=0.823 | -0.09 (-0.60, 0.77)  p=0.802 |
| CXCL10 | 9.7 (0.9) | 9.7 (1.0) | 9.5 (0.7) | 10.1 (1.2) |
|  |  | -0.05 (-0.77, 0.67)  p=0.900 | -0.22 (-0.77, 0.33)  p=0.425 | 0.38 (-0.41, 1.17)  p=0.343 |
| CXCL11 | 3.2 (0.8) | 3.5 (1.2) | 3.2 (0.6) | 3.7 (1.2) |
|  |  | 0.30 (-0.50, 1.11)  p=0.464 | -0.07 (-0.55, 0.40)  p=0.763 | 0.49 (-0.25, 1.24)  p=0.191 |

**Supplementary Table 3. Mean (standard deviation) normalized protein expression values for the chemokines in controls and each of PPA groups in plasma. Mean differences between the PPA groups and controls along with 95% confidence intervals and p values (significant in bold) are shown underneath. N/A = not assessed >80% values were below the lower limit of detection.**

|  | **Controls** | **svPPA** | **nfvPPA** | **lvPPA** |
| --- | --- | --- | --- | --- |
| CCL2 | 10.4 (0.6) | 10.5 (0.7) | 10.6 (0.5) | 10.3 (0.3) |
|  |  | 0.08 (-0.32, 0.97)  p=0.376 | 0.17 (-0.22, 0.56)  p=0.376 | -0.13 (-0.53, 0.26)  p=0.498 |
| CCL3 | 3.8 (1.1) | 4.0 (1.3) | 3.6 (0.4) | **3.2 (0.3)** |
|  |  | 0.19 (-0.72, 1.09)  p=0.688 | -0.24 (-0.77, 0.28)  p=0.362 | **-0.65 (-1.16, -0.13)**  **p=0.014** |
| CCL4 | 7.3 (1.0) | 7.4 (1.2) | 7.4 (0.8) | 6.9 (0.4) |
|  |  | 0.09 (-0.76, 0.94)  p=0.839 | 0.06 (-0.61, 0.72)  p=0.866 | -0.47 (-1.00, 0.62)  p=0.083 |
| CCL7 | 1.8 (0.7) | 1.6 (0.8) | 2.2 (1.0) | 2.1 (0.9) |
|  |  | -0.27 (-0.85, 0.30)  p=0.357 | 0.36 (-0.26, 0.97)  p=0.255 | 0.25 (-0.35, 0.84)  p=0.418 |
| CCL8 | 8.3 (1.1) | **9.1 (0.6)** | 8.7 (1.0) | 8.7 (0.5) |
|  |  | **0.77 (0.17, 1.37)**  **p=0.012** | 0.45 (-0.26, 1.17)  p=0.217 | 0.38 (-0.19, 0.94)  p=0.190 |
| CCL11 | 8.9 (0.5) | 8.8 (0.7) | 9.1 (0.7) | 9.0 (0.6) |
|  |  | -0.05 (-0.54, 0.45)  p=0.849 | 0.20 (-0.22, 0.63)  p=0.349 | 0.13 (-0.25, 0.52)  p=0.501 |
| CCL13 | 4.0 (1.5) | 4.4 (1.1) | **5.0 (1.4)** | 5.0 (1.2) |
|  |  | 0.36 (-0.57, 1.29)  p=0.450 | **1.01 (0.01, 2.00)**  **p-0.047** | 0.95 (-0.01, 1.90)  p=0.052 |
| CCL19 | 10.2 (1.1) | 10.1 (1.1) | 9.8 (1.3) | **9.2 (0.5)** |
|  |  | -0.05 (-0.84, 0.75)  p=0.910 | -0.40 (-1.25, 0.46)  p=0.362 | **-0.96 (-1.52, -0.39)**  **p=0.001** |
| CCL20 | 5.7 (1.0) | 5.3 (0.8) | **5.0 (0.8)** | 5.4 (0.7) |
|  |  | -0.39 (-1.03, 0.26)  p=0.236 | **-0.67 (-1.29, -0.06)**  **p=0.031** | -0.34 (-0.97, 0.28)  p=0.280 |
| CCL23 | 10.3 (0.5) | 10.4 (0.6) | 10.3 (0.4) | 10.2 (0.5) |
|  |  | 0.12 (-0.28, 0.52)  p=0.556 | 0.02 (-0.37, 0.40)  p=0.937 | -0.13 (-0.52, 0.26)  p=0.495 |
| CCL25 | 6.4 (0.7) | 6.8 (0.6) | 6.7 (0.60 | 6.5 (0.7) |
|  |  | 0.36 (-0.14, 0.85)  p=0.155 | 0.23 (-0.24, 0.71)  p=0.321 | 0.03 (-0.45, 0.51)  p=0.903 |
| CCL28 | 1.6 (1.1) | 1.0 (0.5) | 1.1 (0.6) | 1.1(0.5) |
|  |  | -0.54 (-1.10, 0.01)  p=0.055 | -0.52 (-1.09, 0.05)  p=0.075 | 0.47 (-1.02, 0.08)  p=0.097 |
| CX3CL1 | 5.8 (0.5) | 6.0 (0.4) | 5.6 (0.4) | 5.9 (0.5) |
|  |  | 0.14 (-0.21, 0.50)  p=0.413 | -0.21 (-0.54, 0.13)  p=0.223 | 0.03 (-0.32, 0.37)  p=0.878 |
| CXCL1 | 8.7 (1.3) | 9.2 (0.9) | 9.0 (0.8) | 9.3 (1.0) |
|  |  | 0.46 (-0.35, 1.29)  p=0.262 | 0.31 (-0.44, 1.06)  p=0.416 | 0.63 (-0.19, 1.45)  p=0.134 |
| CXCL5 | 9.8 (1.9) | 10.5 (1.5) | 9.8 (0.9) | 10.4 (1.4) |
|  |  | 0.79 (-0.39, 1.96)  p=0.184 | 0.09 (-1.02, 1.21)  p=0.867 | 0.60 (-0.55, 1.74)  p=0.299 |
| CXCL6 | 8.7 (1.5) | 9.2 (1.3) | 9.2 (1.2) | 9.0 (0.4) |
|  |  | 0.54 (-0.47, 1.56)  p=0.291 | 0.48 (-0.41, 1.38)  p=0.290 | 0.34 (-0.35, 1.04)  p=0.335 |
| CXCL8 | 6.2 (1.5) | 6.1 (0.9) | 6.1 (0.9) | 5.7 (0.4) |
|  |  | -0.16 (-1.03, 0.70)  p=0.709 | -0.18 (-1.01, 0.66)  p=0.679 | -0.55 (-1.29, 0.20)  p=0.153 |
| CXCL9 | 7.3 (0.8) | 7.1 (0.7) | 7.1 (0.6) | 7.1 (0.7) |
|  |  | -0.17 (-0.70, 0.36)  p=0.537 | -0.23 (-0.71, 0.25)  p=0.345 | -0.21 (-0.73, 0.30)  p=0.411 |
| CXCL10 | 9.5 (0.9) | 9.5 (1.3) | 9.2 90.8) | 9.1 (0.4) |
|  |  | 0.01 (-0.86, 0.89)  p=0.978 | -0.37 (-0.99, 0.25)  p=0.246 | -0.41 (-0.88, 0.07)  p=0.093 |
| CXCL11 | 9.2 (2.0) | 9.4 (2.1) | 10.0 (1.9) | 10.0 (1.5) |
|  |  | 0.25 (-1.31, 1.80)  p=0.755 | 0.84 (-0.47, 2.15)  p=0.207 | 0.86 (-0.36, 2.09)  p=0.166 |
